# Effect of Surfactant Concentrations on Physicochemical Properties and Functionality of Curcumin Nanoemulsions Under Conditions Relevant to Commercial Utilization

**DOI:** 10.3390/molecules24152744

**Published:** 2019-07-29

**Authors:** Thanida Chuacharoen, Sehanat Prasongsuk, Cristina M. Sabliov

**Affiliations:** 1 Faculty of Science and Technology, Suan Sunandha Rajabhat University, 1 U Thong Nok Rd, Dusit, Bangkok 10300, Thailand; 2 Plant Biomass Utilization Research Unit, Department of Botany, Faculty of Science, Chulalongkorn University, Bangkok 10330, Thailand; 3 Department of Biological and Agricultural Engineering, Louisiana State University and LSU AgCenter, 149 E.B. Doran Building, Baton Rouge, LA 70803, USA

**Keywords:** curcumin, nanoemulsions, surfactant, stability, functionality

## Abstract

Surfactants are used to stabilize nanoemulsions by protecting their physical stability and preventing deterioration of the entrapped bioactive during processing and storage. The effect of surfactant concentration on physical-chemical properties of nanoemulsions with entrapped curcumin, relevant to commercial applications, was addressed in this research. Furthermore, the functionality of nanoemulsified curcumin in terms of lipid oxidation inhibition was determined. Protection against varying pH and thermal treatments was more significant in the nanoemulsions at the elevated surfactant level, but at these high concentrations, the surface charges of the emulsions dramatically decreased under sodium salt addition, which may result in instability over time. Nanoemulsions showed the potential to inhibit malondialdehyde (MDA) formulation by protecting the entrapped curcumin and enhance its antioxidant activity when added to milk. The fortified milk with added curcumin systems had a yellow color compared to the control. The results of the study are critical in choosing the surfactant concentration needed to stabilize emulsified curcumin, and to protect the entrapped curcumin under specific conditions of use to support the utilization of curcumin nanoemulsions as a food additive in different commercial products.

## 1. Introduction

Curcumin is the predominant curcuminoid isolated from turmeric rhizomes. It widely serves as a food ingredient due to its yellow-orange color and its antioxidant activity [1]. Curcumin also possesses various therapeutic properties such as preventing cancer, reducing inflammation, and lowering blood pressure [2], in addition to its ability to inhibit lipid peroxidation [3]. However, applications of curcumin are limited due to its poor water solubility, low bioavailability, and its instability under heating and basic pHs encountered during processing in the food industry.

Curcumin degrades quickly under alkaline conditions [4,5,6] indicated by the fact that the color of curcumin faded when the pH changed from 7.5 to 9 [5]. Similarly, when incubated with 0.1 M phosphate buffer at pH 7.2, 90% of curcumin decomposed within 30 min at 37 °C [4]. Pure curcumin also tended to crystallize out of aqueous acidic solutions (pH < 7) and was subjected to rapid sedimentation [6]. In addition, thermal processing affects the visual color of curcumin as reported by Giménez, et al. [7]. Curcumin undergoes hydrolytic degradation under thermal processing conditions [7,8,9]. The loss of curcumin content rapidly increased with temperature, indicating its instability at high temperatures of 70 and 90 °C. When turmeric was boiled for 10–20 min, 53% of the curcumin was lost.

In order to overcome these limitations, food-grade nanoemulsions have offered several advantages, such as higher optical clarity and better physical stability against particles aggregation, compared to conventional emulsions [10]. Physicochemical properties, stability, and functionality of nanoemulsions depend significantly on the droplet compositions and the ratio of oil, surfactant, and water. Joung, et al. developed curcumin nanoemulsions stabilized by Tween 20. The diameter of the system composed of over 50% aqueous phase with less than 33% and 32% of the oil phase and surfactant content, respectively, decreased from 122 to 90 nm when surfactant concentration increased by three times. The physical stability remained intact for one month at room temperature and up to three months when refrigerated [11]. The work did not address emulsion stability during commercial thermal processing, and exposure to various pH and salt environments. Curcumin loaded in nanoemulsions composed of medium chain triglyceride (MCT), Tween 80, and lecithin were developed by Li, et al. [12]. Chitosan was used as a coating material to prevent the droplets from phase separation. A ratio of MCT oil, lecithin, Tween 80, and distilled water was also proposed at 10:6:4:80. The droplets were 113.93 nm in size and had a size distribution of 0.23 with a negative charge of −36.23 mV due to lecithin presence. Encapsulation efficiency was 95.10%, and it was affected by the amount of surfactant used [13].

Many of these studies worked with high surfactant concentrations, 30 times higher [11,14] compared to concentrations generally used for nanoparticle synthesis [15]. It is important to better understand the effect of surfactant concentration on the stability of nanoemulsions at lower concentrations. Excessive amounts of surfactant may have detrimental effects on the delivery of the entrapped bioactive [16] and can potentially induce undesired characteristics in foods fortified with these components. For example, the ability of nanodelivered curcumin to inhibit lipid oxidation of milk during processing and storage resulting in the formation of undesirable off-flavors and unhealthy compounds can be deterred by excess surfactant used during the fabrication of nanoemulsion systems [17].

Thus, the objective of this experiment was to develop curcumin nanoemulsion systems (Cur-NEs), using MCT oil as a carrier with a combination of lecithin and Tween 80 with a concentration of 0.3%, 3.0%, and 9.0%. Lecithin was selected as a surfactant because it possesses certain beneficial characteristics, such as low toxicity and biocompatibility compared to polymeric surfactants [18]. However, its degradation is particularly pH-dependent. The addition of a nonionic emulsifier, such as Tween 80, as a co-surfactant could possibly enhance the system by preventing aggregation and improving its pH stability. The effect of surfactant concentrations on nanoemulsion systems with entrapped curcumin was assessed in terms of physicochemical stability (mean diameter size, polydispersity index (PDI), surface charge, and curcumin concentration) under processing (pH, ionic strength, and thermal) and storage conditions (4 °C and room temperature). Subsequently, the functionality (lipid oxidation inhibition) of entrapped curcumin and color change when incorporated into milk as a model food matrix were investigated.

## 2. Results and Discussion

### 2.1. Preparation of Cur-NEs

**Figure 1:**
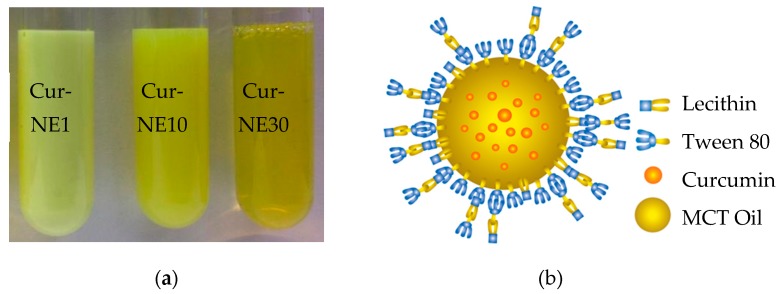
Nanoemulsions formed at three concentrations of surfactant depicted as Cur-NE1, Cur-NE10, and Cur-NE30 (**a**) and schematic structure of curcumin nanoemulsion (**b**).

Curcumin nanoemulsions (Cur-NEs) were synthesized using MCT oil with stabilized surfactants (Figure 1a). The initial curcumin amount of 0.83 mg/mL MCT oil was used for all systems. Lecithin was utilized to stabilize nanosystems and Tween 80, a non-ionic surfactant widely used in foods with its steric effect, was applied as a co-surfactant to prevent the particles from aggregating. The nanoemulsified systems were pictured for visual monitoring immediately after synthesis (Figure 1a). Cur-NE10 and Cur-NE30 were formed at 10- and 30-times surfactant concentration relative to Cur-NE1. The Cur-NE1 solution had a milky character with a light yellowish color due to the bigger size of the emulsions. When higher concentrations of surfactant were applied (Cur-NE10 and Cur-NE30), the average particle sizes decreased resulting in their solutions becoming more transparent (Figure 1a, Table 1). After 15 days of storage in the refrigerator, there were no apparent visual changes in the samples (data not shown).

As seen in Figure 1b, hydrophilic heads of lecithin intertwine with Tween 80, whereas its hydrophobic polypropylene sections connect with the hydrophobic oil phase at the core of the emulsion. MCT oil has the sole role of solubilizing curcumin as indicated by Joung, et al. [11]. The composition and characteristics of curcumin nanoemulsions were measured (Table 1). Of the nanoemulsion systems, at the lowest surfactant concentration tested (Cur-NE1) emulsions had the largest size of 193.93 nm, with a narrow PDI of 0.18, and most negative zeta potential of −62.93 mV. When surfactant concentration was increased by a factor of 10 and 30, the average diameter significantly decreased to 86.68 and 44.39 nm with an increased PDI of 0.19 and 0.23, respectively. Zeta potential values were less negative (−54.27 and −48 mV) at increasing amount of lecithin, because it induced a stronger anionic surface charge resulting in a decreased negative surface charges, similar to data reported by Sari, et al. Sari, Mann, Kumar, Singh, Sharma, Bhardwaj and Athira [14]. As expected, the average diameter of the nanoemulsions decreased with increased surfactant concentration as a result of a larger oil-water interface [11]. Moreover, the combination of lecithin and a hydrophilic Tween 80 reduced the interfacial tension during emulsification and, subsequently, the emulsified droplet size [19]. Encapsulation efficiency was very high ranging from 92.86% to 99.51%. The capability to entrap curcumin increased with the addition of surfactant, as previously reported by Guttoff, et al. [20]. It was concluded that the droplet size, distribution, surface charge, and %EE of Cur-NEs obviously depended on the ratio of surfactant and all systems were significantly stable. While surfactant concentration employed to make Cur-NE30 was commonly utilized for nanoemulsion synthesis in several studies [11,14], these results report 3- and 30-times smaller surfactant concentrations.

### 2.2. Morphology of Cur-NEs

**Figure 2:**
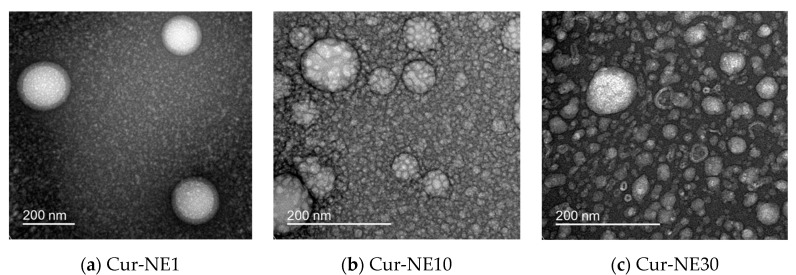
Transmission electron microscope images of Cur-NE1 (**a**), Cur-NE10 (**b**), and Cur-NE30 (**c**) taken immediately after synthesis. The scale bar is 200 nm in each image.

Morphological characteristics of curcumin-loaded nanoemulsions were observed by TEM (Figure 2). The TEM images of curcumin nanoemulsion droplets exhibited smooth surfaces and spherical shapes with a mean particle size of approximately 193.93 nm for Cur-NE1 and smaller droplets at higher surfactant concentrations (86.68 nm for Cur-NE10 and 44.39 nm for Cur-NE30). The sizes of Cur-NEs were consistent with those measured using a zeta size analyzer in the previous section (Table 1).

### 2.3. Physical Stability of Cur-NEs Under Processing Conditions

Physical stability of the freshly prepared curcumin nanoemulsions was investigated when exposed to production stresses that might be affected in practical applications. Average diameter, size distribution, and surface charge of Cur-NEs under pH, ionic strength, and thermal stresses were measured (Table 2). In an alkaline environment, free curcumin degraded due to the de-structured conjugated diene observed by color fading and complete loss of color after 40 h at pH values higher than 7 [5]. The mean diameter and size distribution did not change significantly relative to the control at all pH values for all systems (Table 2).

The negative surface charges of all systems decreased when subjected to acidic conditions, especially for the Cur-NE1 which showed the least negative value (−7.5 mV). The surface charge of the droplets shifted to values less negative at pH values below the isoelectric point of the lecithin (pH 6.7). The results are consistent with the previous study, supporting the poor pH stability of lecithin in an acidic environment [21]. Rao and McClements also found that nanoemulsions were more stable at a neutral pH value, and the droplets increased in size at lower pH values. At these low pH values, the electrostatic repulsion among particles is decreased due to preferential adsorption of hydrogen ions (H^+^) to the droplet surfaces resulting in a lower negative charge and aggregation in highly acidic environments [22]. When the surfactant concentration increased, the charges of Cur-NE10 and NE30 were more negative and, hence, the emulsions more stable (Table 1). Zeta potential values for all systems had lower absolute values in the presence of NaCl, indicating less stability for all emulsions, especially for the Cur-NE1, which had zeta potential close to zero at all three NaCl tested.

Under thermal treatments, size and distribution values of Cur-NEs slightly changed, except for the PDI of Cur-NE30, which increased to about 0.4. Zeta potential values of Cur-NE1 drastically decreased to −14.9 mV and −17.5 mV for both conditions. Tween 80 stabilized nanoemulsions had a smaller zeta potential value after boiling as shown by Sari, et al. [14]. Heating at 80 °C was also found to reduce the zeta potential of lecithin-stabilized nanoemulsions as stated by Guan, et al. [19]. At the higher surfactant concentrations, Cur-NEs were more stable mainly by maintaining the electrostatic repelling force contributed by lecithin and its co-surfactant [23].

### 2.4. Physicochemical Stability of Cur-NEs Under Storage Conditions

Stability of curcumin nanoemulsions was investigated at 4 °C and 25 °C for a period of 15 days to meet the criteria for commercial use. Their attributes were measured in terms of average size, PDI, and zeta potential (Table 3). Phase separation was not observed during storage. After 15 days of storage at 4 °C and RT, the average diameter of Cur-NE1 and Cur-NE10 slightly increased, whereas the size and PDI values of Cur-NE30 changed insignificantly (Table 3). However, the magnitude of the zeta potential of all systems changed as a function of time and temperature. The more surfactant used in the systems, the lower the change. In general, emulsified systems tend to undergo an increase in droplet size attributed to the Ostwald ripening phenomenon. This phenomenon occurring in oil/water emulsified systems is primarily attributable to the solubility of the dispersed oil phase in the continuous aqueous phase. Adding a ripening inhibitor effectively retards the droplet growth over time [24]. Thus, the stability of nanoemulsions is hypothesized to improve when a hydrophilic emulsifier and a lipophilic one are used in combination to stabilize the system. Our results confirmed the effect of a combined surfactant on improving the physical stability of Cur-NEs in terms of size, PDI, and zeta potential.

### 2.5. Chemical Stability of Cur-NEs Under Thermal Processing and Storage Conditions

**Figure 3:**
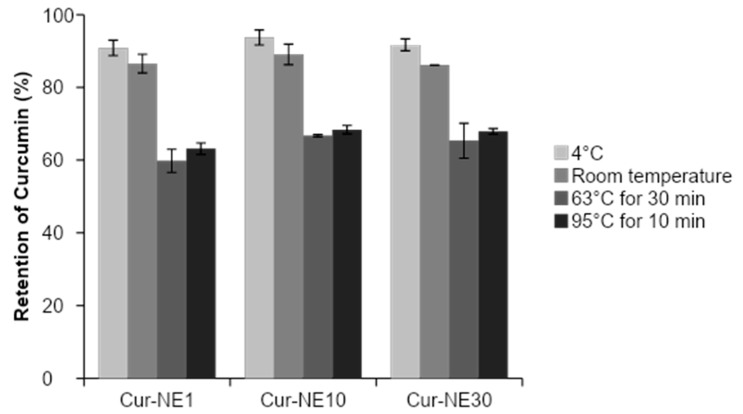
Retention rate of entrapped curcumin loaded in nanoemulsions (NE) systems exposed to refrigerator (4 °C) and room temperature for 15 days, and then during thermal treatments at 63 °C for 30 min and 95 °C for 10 min, respectively.

The degradation of curcumin can occur by autoxidative transformation in the presence of diverse chemicals or when subjected to heat treatment, to degraded compounds such as ferulic acid, vanillin, and vanillic acid [9]. Nanoemulsions stored under room temperature had relatively lower retention rates of curcumin (86 to 89%) than those stored at 4 °C (90 to 93%) (Figure 3). After exposure to thermal treatments, nanoemulsions retained curcumin at rates ranging from 59.77% to 68%. Our results confirmed that the emulsified structures not only maintained its stability but also provided good protection of entrapped curcumin during thermal treatments.

### 2.6. Lipid Oxidation Inhibition of Cur-NEs Fortified with Milk

The assay detects the formation of MDA (malondialdehyde), a secondary metabolite of oxidation formed as a result of the polyunsaturated fatty acid degradation, from lipid oxidative deterioration in lipid-enriched foods. The reaction between the MDA and two molecules of thiobarbituric acid (TBA) forms a pink complex (TBARS) detected by spectrophotometric quantitation at 532 nm of maximum absorption. Cur-NEs were added to commercial milk, and their ability to inhibit lipid oxidation was investigated using TBARS assay. TBARS, as a marker, is suitable to determine the secondary product formed from lipid peroxidation, particularly in milk systems [25,26,27]. The obtained TBARS value is defined as mg MDA equivalent per liter of milk product.

Milk was used as a control, and its total TBARS value was 1.37 mg/L at time zero, and it decreased to 0.98 mg/L and 0.64 mg/L after five and 15 days of storage in the refrigerator, respectively, similar to the range of 1.02 to 1.31 mg/L of MDA concentration from four commercial milk samples reported in a previous publication [28].

Samples of milk with added Cur-NEs investigated at 0, 5, and 15 days had significantly lower TBARS values than the control. At day zero, the TBARS values had ranged from 0.095 to 0.297 mg/L and were statistically significantly different among samples (Table 4). After five days, the TBARS values of all samples decreased and all values were further reduced after 15 days, particularly for samples spiked with Cur-NE10 and Cur-NE30. Results indicated that Cur-NE10 and Cur-NE30 were able to inhibit the MDA formation.

### 2.7. Color Change of Fortified Milk

Curcumin is one of the most commonly used natural food colorant. To better understand color change, colorimetric parameters of milk fortified with these nanoemulsions were analyzed using a chromameter (CM-5, Konica Minolta Sensing Americas, Ramsey, NJ, USA) at 0, 5, and 15 days, and fresh milk was used as a control. The chromatic properties of the fortified milk in terms of lightness (L*), redness (a*), yellowness (b*), chroma (C*), and hue angle (h*) were given in Table 5.

The measured values found for the control were similar to those reported in previous publications [29,30]. With the addition of Cur-NE1, Cur-NE10, and Cur-NE30 to milk, the lightness (L*) of 89.65, 89.66, and 89.70, respectively, increased relative to that of untreated milk (L* = 89.60). The measured a* and b* parameters had the same trend to the lightness.

Chroma (C*), mainly dependent on the yellowness b*, measures color saturation or intensity, while hue angle is used to discriminate among subtle visual color differences. As expected, with the addition of the yellow nanoemulsions, the yellow color of milk increased relative to the control, in a concentration-dependent manner. The intense yellowness parameters of fortified milk decreased slightly compared with that of the untreated milk after storage for 15 days. The high value of h* associated with the low value of greenness (−a*) showed the same trend with the chroma parameter for all of the formulations. The obtained hue angle values were close to the control for Cur-NE1 but were significantly different for Cur-NE10 and Cur-NE30. The greatest color changes were found in Cur-NE10 and Cur-NE30. Thus, fortifying the curcumin loaded nanoemulsions with excessive concentration of surfactant could detrimentally affect the intensity of the milk color.

## 3. Materials and Methods

### 3.1. Materials and Reagents

Curcumin (mixture of demethoxycurcumin and bisdemethoxycurcumin, ≥98%) was purchased from Acros Organic (Geel, Belgium). MCT oil was purchased from Med Lab supply (Miami, FL, USA). Soybean lecithin was purchased from Thermo Fisher Scientific (Waltham, MA, USA), TBARS and Tween 80 were purchased from Sigma-Aldrich (Saint-Louis, MO, USA). All other materials used were of analytical grade.

### 3.2. Preparation of Curcumin Nanoemulsions (Cur-NEs)

Curcumin nanoemulsions were prepared in two stages using the method described by Joung, et al. [11] and Li, et al. [12] with slight modifications. Briefly, 70 mg of curcumin were completely dispersed into 10 mL MCT oil with heating and stirring overnight. The undissolved curcumin crystals were removed by syringe filtration (0.45 μm). Tween 80 and lecithin were dissolved in distilled water which was added to the mixture of MCT oil. The coarse emulsion was then prepared by injecting the mixture into nanopure water containing Tween 80. The ratios of MCT oil, lecithin, and Tween 80 were varied in following compositions: 10:0.18:0.12 (Cur-NE1), 10:1.8:1.2 (Cur-NE10), and 10:5.4:3.6 (Cur-NE30). A fine emulsion was then prepared by microfluidizing the coarse emulsion using a high-pressure homogenizer (M-110P Microfluidizer, Microfluidics, Newton, Newton, MA, USA) at a pressure of 30,000 psi for three cycles. Subsequently, the samples were placed within a dialysis membrane to remove free surfactants for 24 h. After complete dialysis, the samples were kept in the dark, without humidity for further analysis.

### 3.3. Characterization and Morphology of Cur-NEs

Freshly prepared nanoemulsions were analyzed to determine the average particle size, polydispersity index (PDI), and surface charge characteristic using a dynamic light scattering (DLS) instrument at 25 °C. Transmission electron microscope (TEM) was used to image the morphology of curcumin nanoemulsion structures. A single drop of the sample was placed on a copper grid with a carbon film, and a drop of uranyl acetate was added as a negative stain to improve the contrast of the sample prior to TEM analysis.

### 3.4. Determination of Encapsulation Efficiency (%EE) and Curcumin Concentration

Freshly prepared curcumin-loaded nanoemulsions were centrifuged at 10,400× *g* (Allegra 64R, Beckman Coulter Inc., Brea, CA, USA) for 30 min to remove excess curcumin. The precipitate was separated and extracted by 1 mL pure ethanol with vortexing for 5 min. The concentration of curcumin (μg/mL) was calculated according to a standard curve generated by reading at 419 nm of standard curcumin at several concentrations dissolved in ethanol solution using a UV/Vis spectrophotometer (Evolution 201, Thermo Scientific, Waltham, MA, USA). Curcumin concentration was calculated according to a calibration equation (y = 0.148x − 0.0146, R^2^ = 0.999), where y is the absorption at 419 nm and x is the concentration of curcumin (mg/mL). The entrapment efficiency (*EE*) was calculated by the weight ratio of curcumin determined in NE solutions and curcumin initially added using the following Equation (1):

(1) $$EE\;\left(\%\right)\;=\frac{Entrapped\;curcumin\;amount}{Initial\;curcumin\;amount}\;\times \;100$$

The concentration of curcumin was measured to assess its stability under various conditions. Curcumin entrapped in nanoemulsions was subjected to heat and storage conditions, which led to the destruction and mass loss of curcumin. The retention rate (*RR*) of entrapped curcumin is an effective index for the assessment of chemical stability of entrapped curcumin. The solution was extracted with ethanol and vortexed for 5 min. The retention rate (*RR*) of extracted curcumin was determined by measuring the absorbance at 419 nm using a UV/Vis spectrophotometer. The %RR was obtained by using the following Equation (2):

(2) $$RR\;\left(\%\right)=\frac{Cur}{Cur_{0}}\;\times \;100$$

where *Cur*_0_ and *Cur* were the amount of curcumin available at time zero and sequential sampling times, respectively.

### 3.5. Stability of Cur-NEs Under Processing Conditions 

Cur-NE samples were subjected to varying pH conditions (pH 2, 7, and 9), ionic strengths (0.1 M, 0.5 M, and 1.0 M NaCl), and thermal processing (63 °C for 30 min and 95 °C for 10 min) to investigate stability of nanoemulsified curcumin of importance to their commercial utilization. The average diameter, size distribution, and zeta potential were measured after exposing the curcumin nanoemulsions to each treatment. To study the effect of pH on the carriers, the stability of the samples were examined in the pH range of 2 to 9, adjusting the lower pH with 0.1 M HCl solution and higher pH with 0.1 M NaOH solution. After adjustment of the pH, the samples were kept at room temperature for 24 h and, subsequently, characterized with respect to their diameter size, PDI, and zeta potential. For the effect of thermal processing, the samples were exposed to different processing conditions: 63 °C for 30 min and 95 °C for 10 min, and thereafter characterized with respect to their diameter size, PDI, and zeta potential. To assess the effect of ionic strength, the samples were exposed to different ionic strength conditions: 0.1 M, 0.5 M, and 1.0 M NaCl conditions for 24 h and were, subsequently, characterized in a similar fashion.

### 3.6. Stability of Cur-NEs Under Storage Conditions

The physical attributes of Cur-NEs were assessed by storing the samples in the dark at 4 °C and 25 °C for a time period of 15 days. The samples were examined with respect to average size, zeta potential, PDI, morphology, and curcumin retention.

### 3.7. Lipid Oxidation Inhibition by Cur-NEs

Considering the potential of curcumin nanoemulsions for food applications, their ability to act as an antioxidant agent, which prolongs the shelf life of some foods, was determined. Semi-skimmed milk was selected as an aqueous food matrix. The coloring capacity and lipid oxidation of curcumin loaded NE systems added to the milk were investigated at day 0, 5, and 15 of refrigerated storage at 4 °C without light to mimic on-shelf condition with respect to color change. The activity was tested by thiobarbituric acid reactive substances (TBARS) assay [25,29,30].

### 3.8. Color Measurement

The samples were analyzed at room temperature for variations in color using a chromameter (Konica Minolta CM-5, Japan) and results were recorded in CIE Lab system (L*-lightness ranging from black (0) to white (100), a*-color axis ranging from greenness (−a*) to redness (+a*), b*-color axis ranging from blueness (−b*) to yellowness (+b*)). Samples were placed in a cuvette, inserted into a black chamber (provided by Konica Minolta), and connected to the chromameter. The color parameters were determined in triplicate. Chroma (C*) and hue angle (h*) were calculated from a* and b* values using the following equations:

$$C^{*}\;=\;\sqrt{{\left(a^{*}\right)}^{2}+{\left(b^{*}\right)}^{2}}\;\mathrm{and}\;h^{*}=\left(\frac{b^{*}}{a^{*}}\right)\;$$

### 3.9. Data Statistical Analysis

All of the experiments were performed in triplicate (*n* = 3), and the results were reported as the mean ± standard error. Analysis of variance (ANOVA) was performed in SAS version 9.4 (SAS Institute Inc., Cary, NC, USA). The determination of significant differences among the system means was done by Duncan’s multiple range tests. The significance level (*p value*) was set at 0.05.

## 4. Conclusions

In this research, the effect of surfactant concentrations on the formation and stability of curcumin nanoemulsions under conditions relevant to actual production and storage for food applications was evaluated. Nanoemulsions loaded with curcumin were prepared with three different concentrations of surfactants, lecithin, and Tween 80. Increasing the surfactant amount not only decreased the particle size and increased encapsulation efficiency, but also influenced the stability of the droplets and functionality of the entrapped curcumin during processing and storage. After being subjected to processing conditions, nanoemulsion systems seemed to be stable, as measured by size and size distribution. However, negative surface charges drastically decreased under acidic and salt solutions. The stability of the nanoemulsion system with the highest surfactant concentration was highest compared to the other two systems after 15 days of storage. Nanoemulsions with entrapped curcumin possess the potential to inhibit MDA formation, mainly due to protection of the functionality of entrapped curcumin. When milk was fortified with all curcumin nanoemulsion systems, the intense color was significantly changed compared to the control milk after storage for five days. After that, the highest concentration of the surfactant had the most intense color change. Our results concluded that Cur-NE10 can protect physical and chemical stability and functionality of the entrapped compound better than that of the low (Cur-NE1) or high (Cur-NE30) number of surfactants with the latter affecting more the appearance of the fortified milk. Thus, using the proper amount of surfactants can provide the beneficial protection of entrapped curcumin by maintaining the physicochemical stability of the emulsion and functionality of the entrapped bioactive compound under various conditions of use for the benefit of utilizing curcumin nanoemulsions in commercial food products.

## Figures and Tables

**Table 1:** Compositions and characteristics of Curcumin nanoemulsions (Cur-NEs).

	Cur-NE1	Cur-NE10	Cur-NE30
*Composition ratio (%)*			
MCT	10	10	10
Lecithin	0.18	1.8	5.4
Tween 80	0.12	1.2	3.6
*Characteristics*			
Size (nm)	193.93 ± 12.63	86.68 ± 9.58	44.39 ± 3.11
PDI (a.u.)	0.18 ± 0.09	0.19 ± 0.07	0.23 ± 0.02
Zeta Potential (mV)	−62.93 ± 3.22	−54.27 ± 3.90	−48.00 ± 2.83
EE (%)	92.86 ± 0.64	95.38 ± 1.56	99.51 ± 0.75

Results are presented as mean ± SD (*n* = 3). MCT, medium chain triglyceride. PDI, polydispersity index. EE, entrapment efficiency.

**Table 2:** Average diameter, size distribution, and surface charge of Cur-NEs under processing conditions.

	Cur-NE1			Cur-NE10			Cur-NE30		
	Size (nm)	PDI (a.u) ^ns^	Zeta (mV)	Size (nm) ^ns^	PDI (a.u) ^ns^	Zeta (mV)	Size (nm) ^ns^	PDI (a.u)	Zeta (mV)
Control	193.9 ± 12.6 ^a^	0.18 ± 0.09	−62.9 ± 3.2 ^a^	86.7 ± 9.6	0.19 ± 0.07	−54.3 ± 3.9 ^a^	44.4 ± 3.1	0.23 ± 0.02 ^a^	−48.0 ± 2.8 ^a^
*pH*									
2	197.3 ± 0.9	0.19 ± 0.00	−7.5 ± 0.1 ^d^	91.2 ± 2.1	0.16 ± 0.02	−37.2 ± 3.0 ^b^	49.7 ± 0.3	0.29 ± 0.01 ^a^	−23.8 ± 1.6 ^c^
7	191.9 ± 5.8	0.20 ± 0.00	−24.9 ± 4.6 ^c^	90.9 ± 2.7	0.16 ± 0.00	−51.6 ± 1.1 ^a^	46.9 ± 0.8	0.25 ± 0.02 ^a^	−43.6 ± 0.1 ^a^
9	204.4 ± 4.2	0.16 ± 0.01	−51.9 ± 1.4 ^b^	90.6 ± 2.3	0.17 ± 0.01	−50.2 ± 4.4 ^a^	43.9 ± 2.5	0.28 ± 0.00 ^a^	−35.5 ± 6.8 ^b^
*Ionic strength*									
0.1 M NaCl	282.8 ± 6.9 ^c^	0.22 ± 0.02	−2.1 ± 0.3 ^d^	88.7 ± 1.7	0.16 ± 0.02	−8.7 ± 0.2 ^c^	45.7 ± 1.7	0.18 ± 0.01 ^c^	−6.4 ± 0.5 ^c^
0.5 M NaCl	292.5 ± 5.3 ^c^	0.23 ± 0.03	−5.1 ± 0.2 ^d^	86.9 ± 0.4	0.13 ± 0.01	−14.2 ± 1.0 ^c^	44.4 ± 2.7	0.18 ± 0.03 ^c^	−9.4 ± 0.9 ^c^
1.0 M NaCl	220.2 ± 7.8 ^b^	0.20 ± 0.00	−21.3 ± 1.2 ^c^	83.7 ± 2.9	0.14 ± 0.00	−25.2 ± 0.1 ^b^	43.9 ± 2.0	0.22 ± 0.02 ^a^	−18.9 ± 2.8 ^b^
*Thermal processing*									
63 °C (30 min)	197.3 ± 0.6	0.19 ± 0.01	−17.5 ± 0.1 ^c^	81.1 ± 4.7	0.21 ± 0.00	−48.4 ± 1.1	49.2 ± 0.9	0.39 ± 0.01 ^b^	−43.8 ± 1.6 ^a^
95 °C (10 min)	190.9 ± 5.9	0.20 ± 0.00	−14.9 ± 4.6 ^c^	80.9 ± 5.5	0.22 ± 0.02	−50.3 ± 0.1	46.9 ± 0.8	0.42 ± 0.02 ^b^	−43.6 ± 0.1 ^a^

Results are presented as mean ± SD (*n* = 3), data superscripted with a–d in the same rows are significantly different (*p* < 0.05) between control and the treatment. ns, not statistically significant. PDI, polydispersity index. Zeta, zeta potential.

**Table 3:** Average diameter, size distribution, and surface charge of Cur-NEs after 15 days storage at 4 °C and room temperature, RT (°C).

Conditions	Cur-NE1			Cur-NE10			Cur-NE30		
	Size (nm)	PDI (a.u)	Zeta (mV)	Size (nm)	PDI (a.u) ns	Zeta (mV)	Size (nm) ns	PDI (a.u) ns	Zeta (mV)
Day 0 (control)	193.9 ± 12.6 a	0.18 ± 0.09 a	−62.9 ± 3.2 a	86.7 ± 9.6 a	0.19 ± 0.07	−54.3 ± 3.9 a	44.4 ± 3.1	0.23 ± 0.02	−48.0 ± 2.8 a
Day 15									
4 (°C)	211.2 ± 5.1 b	0.08 ± 0.00 b	−51.4 ± 3.1 b	83.5 ± 5.07 a	0.16 ± 0.01	−50.5 ± 2.1 a	46.6 ± 2.5	0.28 ± 0.02	−43.6 ± 5.6 a
RT (°C)	211.4 ± 9.3 b	0.08 ± 0.01 b	−47.3 ± 0.2 b	110.7 ± 8.6 b	0.20 ± 0.03	−35.9 ± 1.1 b	47.1 ± 0.1	0.25 ± 0.05	−20.1 ± 1.8 b

Results are presented as mean ± SD (*n* = 3), data superscripted with a and b in the same type of characters are significantly different (*p* < 0.05) among treatments. ns, not statistically significant. PDI, polydispersity index. Zeta, zeta potential.

**Table 4:** Thiobarbituric acid reactive substances (TBARS) values (mg/L) of entrapped curcumin loaded in NEs in the presence of milk at 0, 5, and 15 days.

Samples	TBARS (mg/L)		
	Day 0	Day 5	Day 15
Control (milk)	1.369 ± 0.020 ^a,A^	0.978 ± 0.014 ^a,B^	0.642 ± 0.027 ^a,C^
Cur-NE1	0.297 ± 0.042 ^b,A^	0.101 ± 0.029 ^b,B^	0.069 ± 0.019 ^b,C^
Cur-NE10	0.209 ± 0.041 ^c,A^	0.087 ± 0.019 ^c,B^	0.028 ± 0.002 ^c,C^
Cur-NE30	0.095 ± 0.035 ^d,A^	0.029 ± 0.039 ^d,B^	0.013 ± 0.001 ^c,B^

Results are presented as mean ± SD (*n* = 3), data superscripted with a–d and A–C show statistically significant difference (*p* < 0.05) due to Cur-NEs and storage times (0, 5, and 15 days), respectively.

**Table 5:** Color parameters of the milk with fortified curcumin nanoemulsions.

Samples	Color Parameters (CIE L*, a*, b* System)				
	L*	a*	b*	Chroma, C*	Hue, h*
*Milk (control)*					
Day 0	89.60 ± 0.17 ^a,A^	−3.02 ± 0.01 ^a,A^	10.22 ± 0.03 ^a,A^	10.66 ± 0.03 ^a,A^	106.46 ± 0.16 ^a,A^
Day 5	89.57 ± 0.01 ^a,A^	−3.06 ± 0.03 ^a,A^	10.18 ± 0.02 ^a,A^	10.63 ± 0.02 ^a,A^	106.73 ± 0.30 ^a,A^
Day 15	89.32 ± 0.02 ^a,B^	−3.22 ± 0.09 ^a,B^	9.16 ± 0.43 ^a,B^	9.71 ± 0.40 ^a,B^	109.37 ± 0.54 ^a,B^
*Cur-NE1*					
Day 0	89.65 ± 0.01 ^a,A^	−3.03 ± 0.04 ^a,A^	10.54 ± 0.03 ^b,A^	10.97 ± 0.03 ^b,A^	106.04 ± 0.36 ^a,A^
Day 5	89.55 ± 0.02 ^a,B^	−3.06 ± 0.02 ^a,A^	10.37 ± 0.03 ^b,B^	10.81 ± 0.03 ^b,B^	106.54 ± 0.23 ^a,A^
Day 15	89.52 ± 0.03 ^b,B^	−3.23 ± 0.01 ^a,B^	10.04 ± 0.02 ^b,C^	10.55 ± 0.02 ^b,C^	107.83 ± 0.16 ^b,B^
*Cur-NE10*					
Day 0	89.66 ± 0.01 ^a,A^	−2.99 ± 0.02 ^a,A^	10.57 ± 0.01 ^b,A^	10.98 ± 0.01 ^b,A^	105.79 ± 0.13 ^b,A^
Day 5	89.55 ± 0.02 ^a,B^	−3.04 ± 0.02 ^a,A^	10.36 ± 0.05 ^b,B^	10.80 ± 0.05 ^b,B^	106.35 ± 0.40 ^b,B^
Day 15	89.53 ± 0.01 ^b,B^	−3.21 ± 0.03 ^a,B^	10.05 ± 0.02 ^b,C^	10.55 ± 0.02 ^b,C^	107.71 ± 0.22 ^b,C^
*Cur-NE30*					
Day 0	89.70 ± 0.01 ^a,A^	−2.95 ± 0.01 ^a,A^	10.63 ± 0.03 ^c,A^	11.03 ± 0.03 ^b,A^	105.51 ± 0.30 ^b,A^
Day 5	89.58 ± 0.01 ^a,B^	−3.04 ± 0.02 ^a,A^	10.42 ± 0.04 ^c,B^	10.85 ± 0.04 ^b,B^	106.26 ± 0.24 ^b,B^
Day 15	89.56 ± 0.03 ^b,B^	−3.19 ± 0.02 ^a,B^	10.09 ± 0.02 ^b,C^	10.58 ± 0.02 ^b,C^	107.54 ± 0.12 ^b,C^

Results are presented as mean ± SD (*n* = 3), values subscripted with a–c and A–C show statistically significant difference (*p* < 0.05) among Cur-NEs and storage times (0, 5, and 15 days), respectively.
